# The Use of English Tube Teeth

**Published:** 1885-10

**Authors:** Lawrence Vanderpant

**Affiliations:** Orange, N. J.


### THE USE OF ENGLISH TUBE TEETH.

BY LAWRENCE VANDERPANT, L. D. S., ORANGE, N. J.

The English firm of dental manufacturers, Messrs. Ash & Sons, have brought to this country a large stock of artificial teeth, some of which would sorely puzzle the average American dentist to utilize. They are called "Tube Teeth," and are almost a counterpart of a natural tooth, excepting that the nerve canal is of larger diameter, passing entirely through the tooth, and it is furnished with a solid, substantial lining of platina.

Until some twenty-five years since, this description was the artificial tooth, principally, if not entirely, used by the European dentist (certainly the English), the majority not even knowing the method of working "American Flat-back Teeth" as they were called.

Now these old-fashioned English teeth, which will doubtless be pronounced by some of our shining lights remnants of old fogyism, are not without their use in modern practice, as I propose to show, and are a very useful adjunct in the office of the practitioner of prosthesis. In a case, for example, where we desire to replace the lost bicuspids and molars by means of a gold or platina plate, they will afford a stronger, broader, and more solid masticating surface; they can be much more accurately fitted to the plate and hermetically united to it by means of the sulphur cement employed in fastening them to the metal pins, so that they are more cleanly in wear than plate teeth of ordinary construction. As only a very small quantity of solder and heat is requisite in mounting, there is no danger of warping the plate, and last, but not least, in case of fracture, tooth or teeth are readily and quickly replaced without the necessity of passing the plate through the fire, which will be an acknowledged advantage. But of more general and especial value would they be likely to prove in crown or pivot work, using them somewhat after the plan of Bonwill or Howe, that is to say, securing, by means of amalgam, cement, or gutta-percha, a pin, pivot, or post into the natural root, and adjusting thereon the artificial tooth crown.

The plan I adopt myself is to first prepare the root and canal, and I find it a saving of much time and trouble to take a small wax model and articulation of the region in process of repair, making a plaster cast therefrom, and to accurately fit and articulate the Tube Tooth to it. It is now absolutely requisite to have "Easy tube sized hard platina or dental alloy wire,"* and proceed to complete the work at the chair. Determine the length of wire requisite for the root and carefully "tin" this portion only.

It may be unnecessary to state here that this is very easily accomplished by dipping the pin into a solution of chloride of zinc, heating it over an alcohol lamp, and rubbing it upon a piece of common domestic tin previously heated.

Two or three small scraps of zinc immersed in dilute muriatic acid (a drachm or so) will conveniently form the chloride.

Roughly and deeply "barb" the root portion of the pin, cut off accurately sufficient to carry the Tube Tooth, and file up to the bifurcation of its lingual surface.

The pin is now securely fixed into the root with amalgam, taking care that its position shall exactly correspond with the tube in the crown.

I should have anticipated the fastening of the Tube Tooth to the pin by advising it to be just sufficiently roughened to catch a strand or two of finest floss silk, previous to fixing it in the root.

It will be within the discretion of the operator to await the thorough hardening of the amalgam previous to securing the crown, or to proceed at once; in either case, it is merely necessary to wind a small quantity of floss silk around the pin and apply a strong mastic solution to the tube, and press the tooth into position by a suitable pair of forceps protected with chamois skin; possibly a judicious tap or two of the protected mallet may make a more perfect joint. The following day an artistic finish may be given to the pin head and lingual surface of the tooth by means of a round headed corundum bur in the engine. I claim for this method many

* The manufacturers supply this wire to exact gauge requisite.

advantages over different methods now in vogue; prominently may be mentioned, great strength, not only from the platina tube, but from the superior hardness and density of the tooth body, and that the crown is fixed without danger of displacement the moment the patient leaves the chair. The objection might be raised that you do not secure a water-tight joint, but I doubt not natural ingenuity will readily surmount this objection.

The weak points in the "Howe" speak for themselves; it is unnecessary to allude to them.

The complicated and expensive work in connection with the gold crown method becomes superfluous in presence of the Tube Tooth plan, and lastly, accident can be so readily provided for that the patient, by furnishing him with duplicate teeth, can rectify it himself.

In writing, I have not had in my mind the replacement of the molar teeth, but a little ingenuity will suggest a means of making Tube Teeth serviceable in such cases.

